# Computational Approaches to Chemical Hazard Assessment

**DOI:** 10.14573/altex.1710141

**Published:** 2017

**Authors:** Thomas Luechtefeld, Thomas Hartung

**Affiliations:** 1 Johns Hopkins Center for Alternatives to Animal Testing (CAAT), Baltimore, MD, USA; 2 CAAT-Europe, University of Konstanz, Konstanz, Germany

**Keywords:** QSAR, machine learning, cheminformatics, molecular descriptor, toxicology

## Abstract

Computational prediction of toxicity has reached new heights as a result of decades of growth in the magnitude and diversity of biological data. Public packages for statistics and machine learning make model creation faster. New theory in machine learning and cheminformatics enables integration of chemical structure, toxicogenomics, simulated and physical data in the prediction of chemical health hazards, and other toxicological information. Our earlier publications have characterized a toxicological dataset of unprecedented scale resulting from the European REACH legislation (Registration Evaluation Authorisation and Restriction of Chemicals). These publications dove into potential use cases for regulatory data and some models for exploiting this data. This article analyzes the options for the identification and categorization of chemicals, moves on to the derivation of descriptive features for chemicals, discusses different kinds of targets modeled in computational toxicology, and ends with a high-level perspective of the algorithms used to create computational toxicology models.

“Big Data is like teenage sex: everyone talks about it, nobody really knows how to do it, everyone thinks everyone else is doing it, so everyone claims they are doing it.”

Dan Ariely, Professor of Psychology and Behavioral Economics at Duke University

## 1 Introduction

The explosive growth of data from biology, physics and chemistry enables the creation and validation of powerful predictive models for toxicological endpoints. Data growth has been paralleled by the creation of new computer science packages for chemical manipulation and model building. The ease of access to chemical data paired with numerous modeling packages has resulted in an increase in the power of computational models for toxicology. “*The goal is*”, as Carly Fiorina, the former CEO of Hewlett-Packard said, “*to turn data into information, and information into insight*.” This is an integral part of the systematic development of the safety sciences (Busquet and Hartung, 2017; Hartung, 2017).

**Fig. 1:**
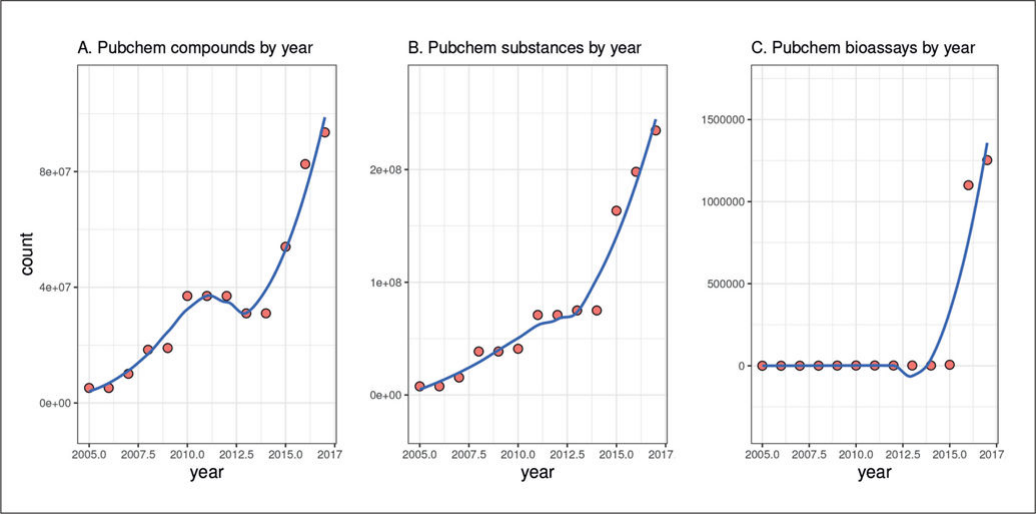
PubChem compound (A), substance (B), and bioassay (C) counts by year Recorded via Wikipedia logs^6^ and Google’s Wayback Machine (Kim et al., 2016b).

Data sources like PubChem show incredible development in the numbers of included compounds, substances and particularly bioassays that they provide in the last few years. In PubChem terminology, a *substance* is a chemical sample description provided by a single source and a *compound* is a normalized chemical structure representation found in one or more contributed *substances*^1^. Figure 1 shows that the number of compounds catalogued by PubChem went from less than 1 million in 2005 to 9.3 million today. The number of substances shows similar growth. The number of bioassays underwent tremendous growth in just the last few years due to the addition of data sources implementing high-throughput systems. But PubChem is just one source of chemical/biological data (Zhu et al., 2016); the number of data sources with overlapping but distinct focuses is also expanding, including REACH data – resulting from the chemical regulation REACH (Registration Evaluation Authorisation and Restriction of Chemicals) in Europe (Luechtefeld et al., 2016a; Hartung, 2010), ChEMBL^2^ – a large-scale bioactivity database for drug discovery (Gaulton et al., 2012), and BindingDB^3^ – a massive catalogue of the binding affinities of chemicals with protein targets (Liu et al., 2007), to name just a few. The combination of these databases provides an important challenge with great potential reward. One example of an effort to combine databases is Chem2bio2rdf^4^, which creates semantic links between chemicals, diseases, symptoms and more (Chen et al., 2010).

The term “quantitative structure activity relationship” (QSAR) is used to describe quantitative relationships between chemical structures and properties. QSARs are built and validated by software development. Development activity can be estimated by querying GitHub^5^ (a web platform for publishing code repositories). Table 1 shows that there are hundreds to thousands of relevant coding repositories for machine learning, statistics, chemistry, biology and, more specifically, cheminformatics and QSARs. These repositories exist in all popular programming languages (Python, Java, R, C, etc.).

The combination of data growth and package development creates new opportunities and dangers for modeling chemicals. Effective models can speed up drug development, improve our environment, protect humans from dangerous exposures, accelerate industrial processes, and more. However, the ease with which these models can be made, paired with their potential commercial impact, creates dangers for development. Care must be taken to create models that can be validated and whose use can be realized in real applications (Fourches et al., 2010; Zvinavashe et al., 2008). Nate Silver, author and founder of the blog FiveThirtyEight, wrote “*When human judgment and big data intersect there are some funny things that happen*”. Nevertheless, toxicological modeling already impacts drug development (Cronin, 2000; Patlewicz, 2006), chemical synthesis (Santana et al., 2006), hazard estimation (Gerner et al., 2004; Fjodorova et al., 2008), and many more real-world applications (Puzyn et al., 2010; Roy, 2017).

### 1.1 OECD QSAR guidelines

The Organization for Economic Co-operation and Development (OECD) recognizes the potential for QSARs to (1) save animal lives, (2) reduce the cost of testing and (3) strengthen chemical regulation (Fjodorova et al., 2008). The OECD created QSAR guidelines in 2004^7^, which form a reference point for many sections in this article (Worth et al., 2005).

1. *A defined endpoint:* QSARs cover diverse physical, chemical, pharmaceutical, toxicological and ecological endpoints. These endpoints can be numeric (e.g., dose/response evaluation), categorical (e.g., chemical classification and labeling), time series (chemical degradation) and others (see Section 4).
2. *An unambiguous and easily applicable algorithm:* The set of algorithms used to derive relationships between chemical structures and endpoints is rapidly growing. Many QSARs use partial least squares or multiple regression. More recently, supervised learning models like neural networks have become popular (see Section 5).
3. *A defined domain of applicability:* Domain of applicability describes the set of chemicals and data a QSAR is built to model. The meaning of domain of applicability changes somewhat depending on the chemicals, data, and modeling techniques employed. This concept works well for QSARs that target chemicals with a specific shared feature (like a model built specifically for methacrylates). Extensions of this concept apply statistical techniques to identify the chemical space that works well for a QSAR (Eriksson et al., 2003). Domain of applicability is discussed in Sections 2 and 5.
4. *Appropriate measures of goodness-of-fit, robustness and predictability:* Justifying the use of a QSAR in commercial or regulatory applications requires an unbiased and valid measure of accuracy. Unfortunately, the rapid growth of statistical software packages and chemical descriptors allows researchers to quickly create new models without fully understanding the pitfalls of underlying features and models. This sometimes leads to a misrepresentation of the accuracy of these models (Fourches et al., 2010). Overfitting and publication bias play a prominent role in the discussion of goodness of fit, which is covered in Section 5.
5. *A mechanistic interpretation, if possible:* A mechanism of action is a well-defined biological interpretation of the means by which a chemical exerts its biological activity. Protein docking QSARs, which model the capacity of a chemical to bind with a receptor, have a clear mechanistic interpretation. QSARs built on chemical similarity or “black box” machine learning methods such as neural networks do not provide a clean mechanistic interpretation. In these latter models, statistical techniques are sometimes derived to help elucidate the modeled mechanisms (Matthews et al., 2009). Section 4 discusses mechanistic interpretation.

### 1.2 Regulatory agency QSAR relevance

Public desire for safety of chemical products regarding human health and the environment has led to more regulatory pressure on chemical industries. Two major manifestations of regulatory pressure are the reform of the Toxic Substances Control Act (TSCA) in the US (Schmidt, 2016) and REACH (Schmidt, 2016) in the EU. Unfortunately, TSCA originally failed to regulate existing commercial substances and set onerous legal constraints for the EPA to take action (Matthews et al., 2009; Schmidt, 2016). The reformed TSCA governs existing commercial substances as well as new substances. As a result, more than 80,000 commercial substances will need to be evaluated, which is far more than the EPA can evaluate using traditional methods. To accelerate chemical evaluation, the EPA is already using QSARs to evaluate ecologic and some health hazards (Malloy and Beryt, 2016).

The REACH legislation requires companies to submit detailed reports including ecological and toxicological information on all compounds used in excess of one ton per year. This legislation came into force in 2007 and has sparked similar efforts in China, Turkey, Japan, Taiwan, South Korea, Malaysia, USA, and India (Matthews et al., 2009; Silbergeld et al., 2015). To satisfy REACH and the parallel Classification, Labeling and Packaging (CLP) legislation, companies must generate United Nations Globally Harmonized System (GHS) classification and labeling for each chemical (Winder et al., 2005). These labels are alphanumeric identifiers (e.g., H317 = skin sensitization), which form ideal “well-defined” endpoints for QSARs. Depending on the chemical and its usage level, some substances can satisfy reporting requirements with QSARs and chemical similarity approaches (termed read-across) (Patlewicz et al., 2014; Ball et al., 2016). QSARs used in such reporting cost a fraction of the replaced animal test and present a strong economic case for many companies (Scholz et al., 2013).

### 1.3 The academic QSAR community

Academic pursuit of QSARs provides research into and education for theory, software development, and evaluation of tool quality as well as potential impact in diverse applications. Development of many open source chemistry packages widely used in commercial and regulatory applications rely on academic effort including the Chemistry Development Kit (Willighagen et al., 2017), Chemminer (Cao et al., 2008), OpenBabel (O’Boyle et al., 2011), and RDKIT (Landrum, 2013).

A PubMed query of “QSAR”, which is a controlled MeSH term, reveals 15,328 publications as of July 20, 2017^8^. There are more than 5,000 unique authors in the QSAR field.

**Fig. 2:**
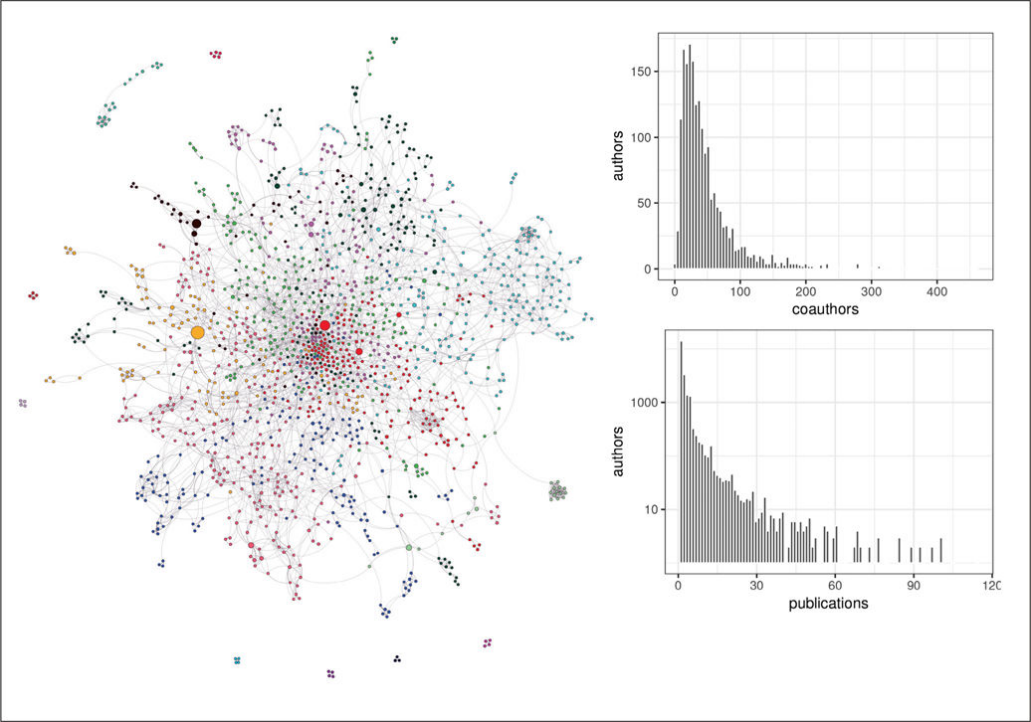
Co-authorship graph for the QSAR field Left: Force layout graph of co-authorship of publications matching the “QSAR” PubMed query. Authors are nodes and edges are drawn between those who have co-authored a publication. Upper right: Number of authors with x number of coauthors. Lower right: number of authors (log scale) with number of publications matching “QSAR” PubMed query

Figure 2 on the left shows that the QSAR field is a tightly knit group. The nodes in the graph represent authors with more than 10 publications and the edges identify authors who have published more than 2 articles together. The graph is strongly connected with only a few dozen authors not part of the central connected component.

## 2 Chemical identifiers and their problems

Computational models that derive chemical activity from chemical structure are called QSARs. QSARs reduce chemicals to constituent atoms, bonds, charges and conformations (3 dimensional shapes). Additional properties can be constructed from these basic building blocks. A QSAR purist asserts that chemical activity is ultimately a function of chemical structure.

The first step in any model design is identification of what one seeks to model. This philosophical sounding point has surprising practical relevance in cheminformatics, where curating chemical sets to remove duplicates and identify distinctly encoded but matching chemicals is considered a paramount initial step (Fourches et al., 2010; Oprea et al., 2003). Chemicals have surprising means to avoid unique/unambiguous identification and the categorization of chemicals can be almost arbitrary. This chapter reviews the basics behind naming and categorizing chemicals based on their structure.

### 2.1 Chemical identifiers

**Fig. 3:**
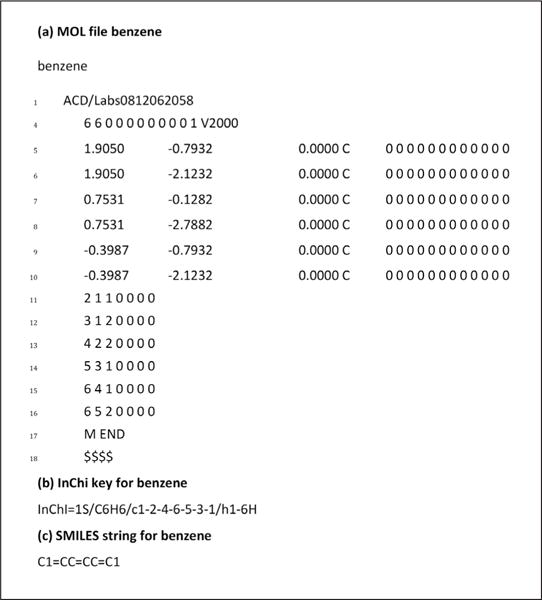
Examples of chemical identifiers (a) MOL file for benzene on Wikipedia^11^, (b) InChi key for benzene^12^ and (c) SMILES string for benzene (Weininger, 1988)

Well-curated data creates stronger models that can have more applications. One principle of database design is the use of chemical identifiers. Unfortunately, the world of small molecules suffers from a naming problem. It is difficult to combine data from databases that reference chemicals by common names or use internal identifiers. Issues in data quality also have a pernicious ability to pollute databases that seek to integrate data between different sources (Oprea et al., 2003). Some instructive and commonly used chemical identifiers include MOL files, InChi keys, and SMILES notations (Fig. 3).

– *MOL files:* a MOL file defines the atoms, bonds and conformation of a chemical completely. Figure 3 shows an example MOL file for benzene: the top 3 lines are header information, line 4 defines number of atoms and number of bonds, lines 5-10 (for this atom) describe atom x,y,z locations (in angstroms) and charges. Lines 26 through 31 describe bonds (first atom, second atom, bond type) (Dalby et al., 1992). MOL files are a unique description of a specific conformation of a compound. Because MOL files are unique at the level of conformation, they make a very strong identifier.
– *InChi:* InChi is supported by the International Union of Pure and Applied Chemistry (IUPAC). It is an algorithm that generates a unique single-line string for a compound. It captures atom, bond and charge information but does not capture atom locations. InChi keys use a canonicalization algorithm that guarantees that the same structure will always be mapped to the same string^17^. While InChi keys do not support exact conformation, they do support stereochemistry and can discern between stereoisomers.
– *SMILES:* SMILES (Simplified Molecular Input Line Entry System), the most widely used chemical identifier (Heller et al., 2015), encodes chemical structures as single-line strings. The SMILES encoding breaks down into four separate encodings depending on the desired resolution (Willighagen et al., 2017):
  - *generic:* Also termed non-canonical. The same structure can have different SMILES strings which order atoms differently.
  - *unique:* Also termed canonical. The same structure will always output the same SMILES. Canonical SMILES do not handle isotopes or stereochemistry.
  - *isomeric:* Non-canonical. The same chemical can have multiple SMILES strings with different atom orders. Isotope and stereochemistry is encoded.
  - *absolute:* Canonical encoding that handles isotope and stereochemistry.
  The flexibility of SMILES makes it a popular format. It is easy to create a SMILES encoding for every chemical in a set without knowing details about the chemicals. This is a strength in that identifiers can always be generated, but it is a weakness because non-expert usage can lead to loss of information and potential identifier collisions and non-uniqueness. Additionally, the lack of a consensus supporter for the SMILES algorithm leads to a variety of implementations in different packages (Heller et al., 2015). DAYLIGHT chemical information systems^9^ gives a comprehensive description of each kind of SMILES. For this review, it is sufficient to understand that SMILES have differing levels of resolution and that absolute SMILES must be used to provide a unique and unambiguous chemical encoding (up to the level of conformation).
– *Non-algorithmic identifiers:* MOL files, InChi keys, and SMILES strings are all algorithms that derive identifiers from chemical structure. There are many non-algorithmic identifiers that include common names, vendor names, and internal database names (such as PubChem’s CID). When databases use non-algorithmic identifiers, this can lead to poor data quality and more difficulty integrating data sources. CASRN (Chemical Abstract Service Registry Number)^10^ is an important example of a non-algorithmic identifier. CASRN is supported by the American Chemical Society and provides a naming service for chemicals and substances (combinations of chemicals). It is widely used in publication and analysis of chemicals.

### 2.2 Problems of chemical identifiers

#### The problem of identifier collisions (many chemicals → one identifier)

When different chemicals map to the same chemical identifier, this results in an identifier collision. Collisions are a problem for integrating databases and compiling chemical information. This can happen when a common chemical name actually refers to many different but related chemicals (e.g., lactic acid has three associated CAS numbers). An ideal experiment observes a property for a specific chemical structure. That observation is stored in databases by pairing a chemical identifier, an observation identifier (such as a property name), and a value. But if the identifier can be mapped to multiple chemicals, users of the database cannot determine which structure was really used in a given experiment. Identifier collisions are actually quite common in practice. Although collisions generate significant concern for experimental reproducibility (Fourches et al., 2010), their impacts on QSARs can vary from severe to non-issue. Identifiers like generic SMILES, which may result in identifier collisions, impose strong constraints on the chemicals that may collide. Chemicals with structures that are similar enough to map to the same generic SMILES will often have similar properties. This is the chemical similarity hypothesis and it is reviewed in Section 5. A more critical problem for computational models is when the same chemical can map to different identifiers. In an analysis of the prevalence of these collisions, Akhondi et al. (2012) found that stereochemistry accounted for the majority of identifier problems and that this was a common problem even in large databases. This analysis can be done with the use of FICTS rules to modify structures by (F) removing small organic fragments, (I) ignoring isotopic labels, (C) neutralizing charges, (T) generating canonical tautomers, (S) ignoring stereochemistry.

#### The problem of non-uniqueness (one chemical → many identifiers)

When one chemical can be mapped to multiple identifiers, there is a non-uniqueness problem. QSARs use training data in the form of observations on chemical identifiers. When data is duplicated or attributed incorrectly, QSARs suffer bias. If chemicals that are in fact the same map to different identifiers, then they may bias an otherwise well-diversified dataset. Non-uniqueness has more critical effects when combining data from different sources. If a drug has one name in DRUGBANK^13^ and a different name in ChEMBL, then training data built using both sources will fail to attribute properties correctly to this chemical. It is important to consider that QSARs do not operate directly on chemical identifiers. Instead, QSARs use feature vectors for chemicals, these vectors describe important characteristics of chemicals and define the resolution at which a QSAR can evaluate a chemical. If a QSAR uses a single feature, the water octanol coefficient for example, then chemicals that map to the same water octanol coefficient are indistinguishable to the QSAR. Because chemical identifier mistakes are non-random, their impact on a QSAR can vary from severe (many duplicates, misattributions) to minor (incorrect identifiers that map to the same feature vector). This subject is revisited in Section 5.

#### The problem of mixtures

QSARs are constructed from experiments that assign properties to chemical feature vectors (built from chemical structures). In reality, it is very difficult to causally link a specific chemical to a specific property. Experiments are done in the context of an environment that can include other chemicals such as the solvent used to dissolve the target chemical and transformations of the target chemical. Attributing risk to a specific compound is not only difficult but also not what is ultimately needed. An ideal model determines risk in real world scenarios, where compounds never exist in a vacuum. This is why, for example, pesticides are retested if even a relatively inert constituent is changed (Lydy et al., 2004).

Identifying mixtures relies on non-quantitative methods including the chemical abstract service registry (CASRN numbers) and PubChem’s substance identifier (SID)^14^. These identifiers can cause problems when integrating data between databases.

Mixtures are important in the context of purity as well. Adverse reactions to drugs can be the result of impurities. These impurities are the target of their own QSARs (Valerio and Cross, 2012) show for example an effective mutagenicity QSAR for pharmaceutical impurities). Training data for QSARs is impacted when observations are the result of impurities rather than target compounds. To handle impurities, some databases require curation of chemical data guaranteeing high purity. One example of this is the *Bundesinstitut für Risikobewertung* (BfR – Federal Institute for Risk Assessment) database in Germany, which requests for purity in excess of 95% for all chemicals (Gerner et al., 2004). Careful curation of chemical purity is an important part of data maintenance; however, it is neither necessary nor sufficient to determine a causal link between chemicals and their observed properties.

One reason for the insufficiency of chemical purity to determine causal linkages is the transformation of chemicals in different exposure environments. Thus, a negative result for a pure chemical does not rule out the possibility of transformation to a more hazardous compound. Efforts to computationally model chemical transformations are sometimes incorporated directly into QSARs. These models can be considered integrated testing strategies, which first break a target compound into all its possible transformations and then seek to model the whole set (Dimitrov et al., 2005). QSARs taking this approach include CATABOL (Dimitrov et al., 2005), TIMES (Mekenyan et al., 2004) and DEREK (Ridings et al., 1996).

### 2.3 Chemical categorization

Chemical categorization is useful in the creation and validation of QSAR models. Narrow QSARs are designed specifically for a single “group” of chemicals. This group is termed the model’s “domain of applicability” (Sushko et al., 2010) and its definition is required in the OECD QSAR guidelines (Fjodorova et al., 2008). Chemicals can be grouped into disjoint sets or into hierarchical groups. These groups can be expertly defined, algorithmically defined, or use enrichment analysis to combine expert definitions and algorithmic definitions.

#### Expert-defined groups

Expert-defined chemical groupings group chemicals by shared properties. These shared properties can take on diverse forms such as biological roles, environmental action, or shared functional groups. PubChem provides many of these categorizations, for example:

– *ChEBI*^15^ *biological roles:* an ontology defined on chemical biological roles like “glutathione depleting agent” (Hastings et al., 2013).
– *FDA pharmacologic classification:* The FDA provides drug classes defined by chemical structure, mechanism of action, and physiological effects (FDA, 2009).
– *MeSH terms:* The NCBI medical subject headings (MeSH) terms are a powerful controlled vocabulary defining many different subjects. In the context of chemical grouping, these terms are defined based on concepts such as structure, biological effect, pharmaceutical preparations and more.

The last example, NCBI Chemical MeSH Terms for Chemicals and Drugs, is a hierarchy of keywords, a subset of which groups chemicals by shared functional groups and atomic characteristics. Examples of this grouping include “aldehydes”, “alcohols” and “hydrocarbons”, all of which are groups of chemicals with shared substructures or atom types.

A specific example of this is the MeSH grouping for “thiazolidenedione”^16^. This MeSH term is defined as:

D02.886.675
D03.383.129.708
THIAZOLES with two keto oxygens. Members are insulin-sensitizing agents, which overcome INSULIN RESISTANCE by activation of the peroxisome proliferator activated receptor gamma (PPAR-gamma).

**Fig. 4:**
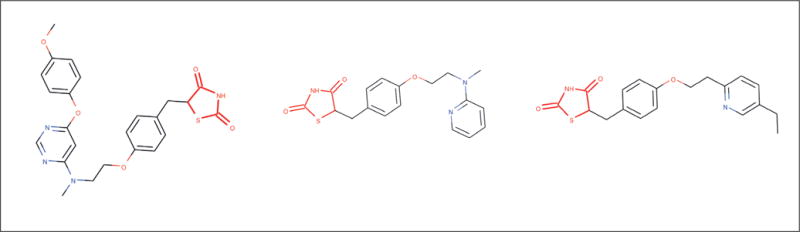
Example thiazolidenediones structures Three example thiazolidenediones (lobeglitazone, rosiglitazone and pioglitazone) are depicted with their matched thiazolidenedione substructure colored in red.

The tree number “D02.886.675” identifies parent terms to thiazoles, which are D02 = organic chemicals and D02.886 = sulfur compounds. MeSH terms can belong to multiple MeSH trees as identified by the secondary tree number “D03.383.129.708”, which corresponds to heterocyclic compounds [D03], 1-ring [D03.383], azoles [D03.383.129]. These parent terms give context for the definition of thiazoles as organic sulfur compounds that are 1-ring heterocyclic azoles. The verbal definition given below the tree numbers identifies thiazolidenediones as thiazoles, which means they contain a thiazole group. This definition is visualized in Figure 4 by highlighting the red thiazole group in three thiazoles.

**Fig. 5:**
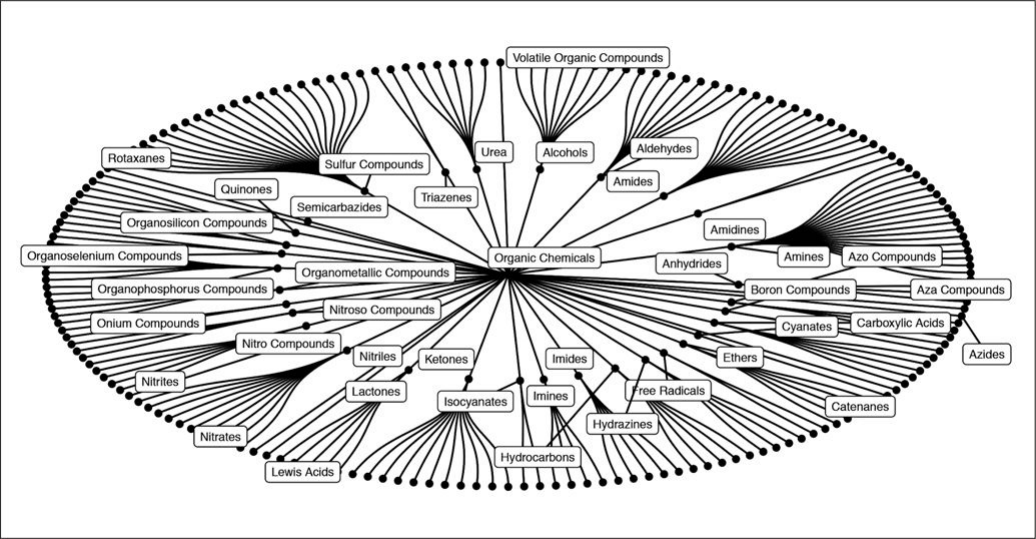
MeSH hierarchy for the MeSH term D02 – “organic chemicals” This is one example of a chemical ontology. Organic chemicals are grouped together under D02, organic chemicals with a sulfur atom are grouped under “sulfur compounds”. Compounds and MeSH categories can have multiple parents, e.g., a sulfur compound can be inorganic or organic. Multiple inheritance is not visualized here.

One common grouping shared by MeSH is the divide between organic and inorganic chemicals. The hierarchy under “organic chemicals” is shown in Figure 5. The parent term “organic chemicals” is divided into many child terms, which are divided into more child terms. This is visualized as a tree and cut off at 3 levels for visualization purposes.

As we have seen, some hierarchical groupings support multiple inheritance, where a single concept/compound can be shared between multiple groups. For example, the left compound in Figure 4, lobeglitazone, is both a thiazolidenedione and a pyrimidine (it has a pyrimidine heterocyclic ring substructure). Ultimately, expert systems define their groupings to help humans think about the chemicals within a group. Inorganic and organic chemistry are defined because chemicals in each group are expected to behave differently and have relevance in different contexts. Expertly defined chemical groupings are a good way to attach human meaning to groups of chemicals. However, expert systems suffer when they do not provide complete definitions of the inclusion/exclusion criteria for chemicals. For example, the given thiazolidenediones definition does not make clear whether non-insulin sensitizing agents with two keto oxygens and a thiazole group are thiazolidenediones. Quantitative approaches to chemical grouping do not suffer this problem, but also lack the same human relevance that can easily be achieved in expert systems.

#### Quantitative grouping

Non-hierarchical quantitative clustering usually involves two steps:

1. Users select features that are relevant to a specific interest such as the presence/absence of chemical substructures (Barnard and Downs, 1992) or biological features such as binding affinities to different receptors (Zhou et al., 2014).
2. Users employ an algorithm that groups “similar” feature vectors. These algorithms usually explicitly (k-means) or implicitly (self-organizing map) employ a distance metric such as Euclidean distance between feature vectors.

Hierarchical quantitative clustering works by comparing each chemical and iteratively creating groups of compounds. Once the first partitioning is done (assignment of chemicals to distinct groups), partitioning is repeated on the groups of compounds. This requires that a distance metric be definable on both the feature vectors describing the compounds and on groups of compounds.

Quantitative grouping is reviewed in more detail in Section 5. It is mentioned here to make the reader aware of alternatives to expert defined chemical groupings. Expert defined chemical groupings and quantitative groupings are not entirely distinct as quantitative approaches can use expertly defined features (such as MeSH term assignments).

#### Example application

**Fig. 6:**
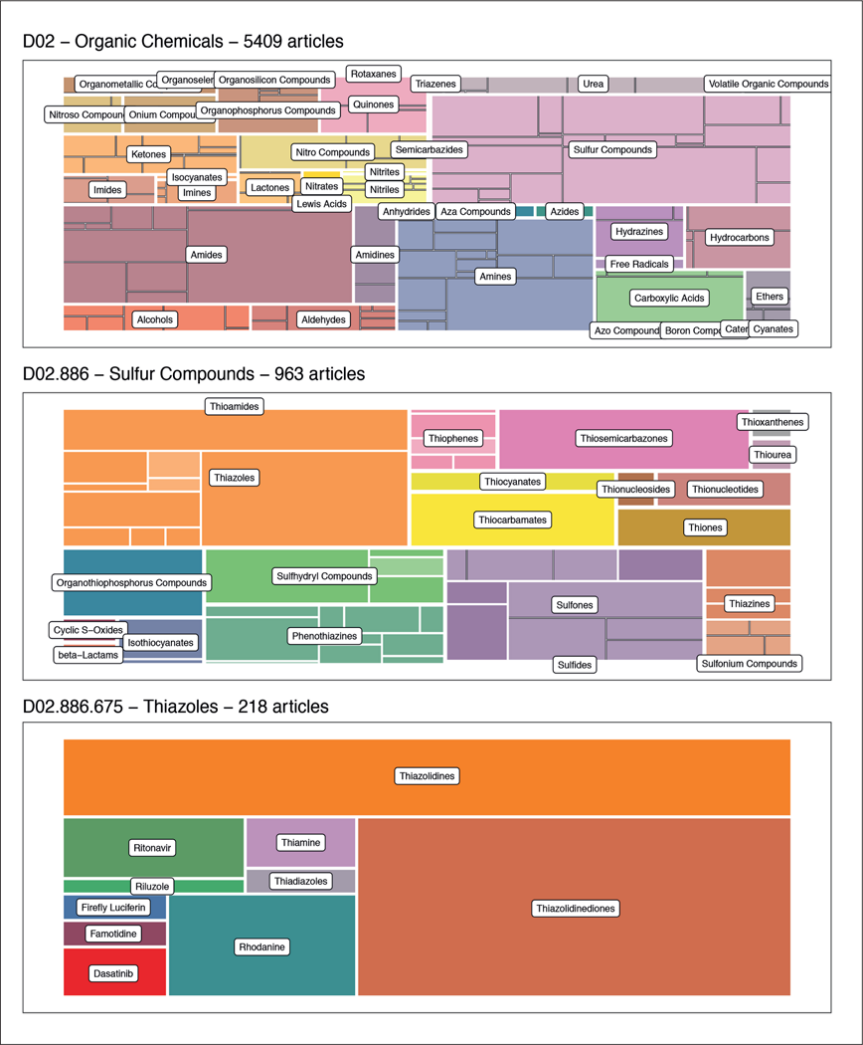
Chemicals categorized under the MeSH term D02 – “organic chemicals” Each block is named by a corresponding MeSH term and sized according to the number of articles containing the MeSH term or a child MeSH term. Blocks are subdivided by their child MeSH terms (up to 3 layers deep).

In the context of QSARs, one might ask which compounds have been modeled most often in publications. NCBI labels articles with MeSH terms corresponding to chemical groupings (described above) and also captures the concept of QSAR (MeSH term D021281^17^). To answer this question, all articles matching QSAR in Pubmed (15,000 articles) were reduced to those mentioning MeSH term D02 “organic chemicals” (5,000 articles) and the prevalence of different subgroups of organic chemicals in item (2) corpus (Fig. 6, top) was determined. Figure 6 (top) is a treemap of all the 5,409 QSAR articles with the “organic chemicals” MeSH term. Chemical categories that are children of the “organic chemicals” MeSH term are labeled in the top treemap. Larger subgroups are chemical groups that are more prevalent. Sulfur compounds are the most commonly labeled subgroup of organic chemicals in QSAR papers. From this it can be surmised that sulfur compounds are commonly modeled in QSAR publications. This process can be repeated for all sulfur compounds. 963 articles remain after filtering out all articles that are not labeled with “sulfur compounds”. In these articles, the sulfur compound subgroup thiazoles is the most common subgroup (Fig. 6, middle) although other prominent subgroups like the sulfones and organothiophosphorus compounds (which have no child groups) are also prevalent. The subgroup thiazolidenediones are the most commonly referenced child of the “thiazole” group in the 218 publications labeled by thiazole (Fig. 6, bottom). They are also a MeSH term “leaf” node, meaning that there are no subcategorizations.

The value of chemical categorization is apparent in this toy example as it lets us find groups of chemicals that are targeted by model developers. Since thiazolidenediones appear to be a relevant target of QSARs, we find the terms that are more prevalent for this group (papers matching QSAR and thiazolidenediones) relative to all QSAR publications. Thiazolidinedione QSARs model humans much more often than most publications about QSARs. They employ molecular models more often, and use techniques like molecular docking simulation, molecular conformation, and x-ray crystallography. They also investigate antineoplastic/antitumor activity more often, which links to the “insulin-sensitizing agents, which overcome INSULIN RESISTANCE by activation of the peroxisome proliferator activated receptor gamma (PPAR-gamma)” MeSH term definition of thiazolidines.

This example explored the ability for chemical categorization to identify targets of QSAR development. It went further and identified differential concepts associated with thiazoles. This analysis termed “enrichment analysis” here, is the heart of all chemical modeling. Algorithms seek to find discerning features of chemicals by correlating these features or functions of these features to biological activities. MeSH headings themselves have been used for computational modeling: Keiser et al. (2007) correlated MeSH headings to biological activity class and successfully identified that methadone, an NMDA antagonist, is also an M3 muscarinic receptor antagonist (a previously unknown property) (Nigsch et al., 2009).

## 3 Features of chemicals

The second step in model development, after identifying chemicals, is identifying descriptive features for each chemical. For example, in our earlier work to predict potency of skin sensitization (Luechtefeld et al., 2015), we used CAS numbers (see Section 2) to identify chemicals. The descriptive features, also termed the “manifest”, included: CD86 – an *in vitro* assay for dendritic cell activation, DPRACys – the direct peptide reactivity assay for cysteine which measures the ability of chemicals to bind to cysteine, DPRALys – the same as DPRACys but for lysine, and KEC1.5 and KEC3 – the KeratinoSens 1.5-fold and 3-fold *in vitro* luminescence measure for KEAP1-NRF2 activation. For this example dataset, we sought to define a function for the target feature LLNA (local lymph node assay), i.e., *F*(*CD86*, *DPRACys*, *DPRALys*, *KEC*1.5, *KEC*3) = *LLNA* or a conditional probability distribution *P*(*LLNA*|*CD*86, *DPRACys*, *DPRALys*, *KEC*1.5, *KEC*3).

The QSAR hypothesis is that all chemical activity is a function of chemical structure – the bonds and atoms found within the molecule (Hoffmann and Hartung, 2006). While this hypothesis may be true, machine learning requires large amounts of data to fit parameters and build probabilistic relationships. Sometimes structural information alone fails to provide the magnitude of data required for strong predictive models.

Thus, QSAR models may use biological or physical properties of chemicals that may be much more information dense than structural features. The features in our study (Luechtefeld et al., 2015) were selected from over 1000 structural and biological features as the most “important” features for skin sensitization modeling (feature selection is discussed in Section 5). Biological features like these can outperform structural features due to their mechanistic relationship with the target (in this case skin sensitization).

To collect more information, model builders look for all predictive features that can be aggregated and assigned to chemical structures. In principle, model builders can use any property with direct or indirect causal relationships with an endpoint. One reason for the common use of structural features is the ease of their assignment. Once a chemical structure is known, structural features can be generated for free (or at least at the cost of electricity). Other features, such as biological assays, can be expensive and slow to collect. A few common chemical descriptors include:

1. *Molecular graph:* Chemical structures are treated as a mathematical graph with atoms as nodes and bonds as edges. Features are derived by different methods of traversing the graph (Leach and Gillet, 2007).
2. *Quantum mechanical:* Chemicals are modeled via the molecular wave function and features related to bond strength, electron distribution, and electromagnetic force are derived (Puzyn et al., 2008).
3. *In vivo and in vitro:* When building models for human hazards, toxicologists typically use animals or cells. For example, the mouse LLNA exposes mice to a chemical and measures response in lymph nodes (Gerberick et al., 2001). The result of this *in vivo* assay is a feature used in modeling human skin sensitization. Due to their expensive nature, *in vivo* endpoints are often the target of their own models.
4. *Molecular biology:* Microarrays, proteomics, metabolomics, RNA-sequencing, genetic sequencing, methylation bisulfite sequencing and other means to assess genetic impact provide rich features for describing the impacts of chemical exposure (Low et al., 2011).
5. *Semantic:* Semantic databases capture relationships between entities such as *Chemical A* → *is similar to* → *Chemical B.* When chemicals are present in a semantic database, their relationships to other entities can be used as chemical features. Chen et al. (2012) use metapathways as features to describe chemicals. For example, when modeling ligand-receptor relationships, the metapathway *Chemical A* → *is similar to* → *Chemical B* → *is ligand of* → *receptor i* is a useful descriptive feature for predicting whether chemicals are ligands of a given receptor (Chen et al., 2012; Fu et al., 2016).
6. *Ensemble:* The output of models can be used as inputs to other models in *ensemble* machine learning methods (Leach and Gillet, 2007).

The recent growth in magnitude and diversity of experimental data for these features is one reason for the recent success of QSAR modeling (Cherkasov et al., 2014).

In this review we discuss physics based and molecular graph descriptors in more detail due to their prevalence in the field (Voutchkova et al., 2010). The book “*An Introduction to Chemoinformatics*” by Leach and Gillet (2007) is an excellent introductory source of cheminformatics features discussion (and other cheminformatics knowledge).

### 3.1 Molecular graphs

Chemicals can be modeled as graphs, where nodes are atoms and edges are bonds. Treating a molecular structure as a graph allows graph traversal algorithms to characterize the structure. These traversal algorithms are broadly called vectorizers because they map structures to numeric vectors. Most cheminformatics packages implement simple functions for deriving different kinds of vectorizers. Specific graph traversal algorithms include hashed and Morgan fingerprints, topological indices and dictionaries.

#### 3.1.1 Hashed fingerprints

Hashed fingerprints map a molecular graph to a set of subgraphs and map those subgraphs into integers. Hashed fingerprints are able to generate features for any set of chemicals without requiring an *a priori* definition of each subgraph of interest. From a practical perspective, hashed fingerprints can require a two-pass algorithm where first all the subgraphs present in a chemical set are aggregated and then vectors are generated for each chemical.

Hashed fingerprint molecular subgraphs are usually built via a graph traversal. Python code snippet shows how to generate Morgan fingerprints using RDkit (Landrum, 2013). Line 5 shows that a list of integers defines the numeric vector for sarin (which has SMILES code “FP(=O)(OC(C)C)C”):

1. from RDkit import Chem
2. from RDkit.Chem import AllChem
3. sarin = Chem.MolFromSmiles(“FP(=O)(OC(C)C)C”)
4. AllChem.GetMorganFingerprintAsBitVect(sarin, radius=2,nBits=2048)
5. > res[0] = [192,1,2017,486,1057 …

Hashed fingerprints are usually high dimensional (there are many possible subgraphs) and sparse (any specific chemical lacks most of the catalogued subgraphs). To store hashed fingerprints more efficiently, most cheminformatics packages write hashed fingerprints as sparse vectors that only store information on the subgraphs present for a given chemical and assume a default value of 0 or false for subgraphs that cannot be derived from a parent chemical. The fingerprint in line 4 is a sparse vector (sometimes represented by a bit set).

#### 3.1.2 Morgan fingerprints

**Fig. 8:**
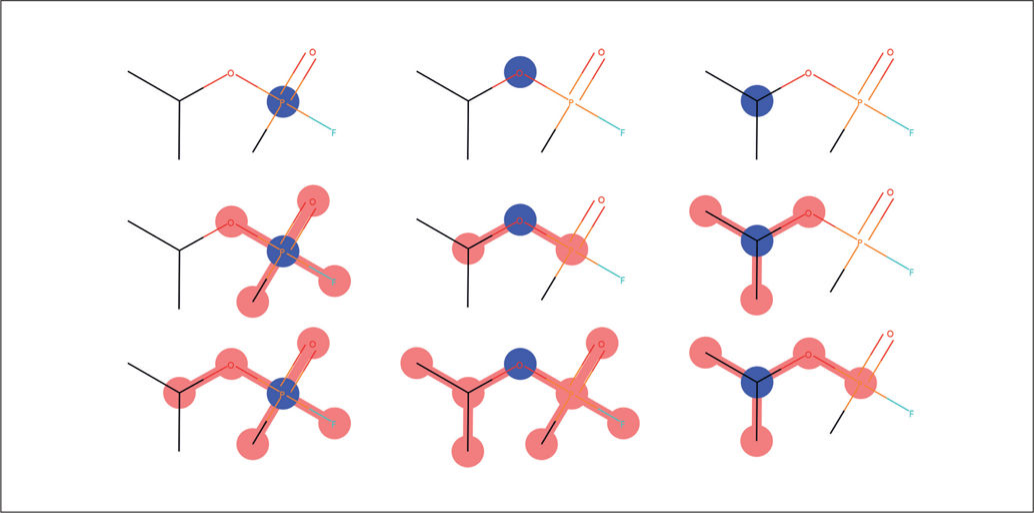
Morgan Fingerprint generation Common central atom colored in blue on columns. First row = 0 neighbors. Second row = 1 neighbor. Third row shows 2 neighbors. (Landrum, 2013)

Morgan fingerprints (also circular fingerprints) are an example of hashed fingerprints. They take a central atom and then select all the atoms within a radius of that central atom. This selection of atoms is mapped to an integer via a hashcode. Morgan fingerprints sometimes simply encode the presence/absence of different subgraphs and sometimes actually count the number of times each subgraph occurs in a chemical. Figure 8 shows the subgraph creation process for Morgan fingerprints. In the top row we see all the subgraphs of radius 0 (0 neighbors selected around a central atom). The second row shows subgraphs of radius 1. The maximum radius is a parameter selected by the user.

#### 3.1.3 Topological indices

Topological indices are numeric functions of graphs. A topological index (TI) transforms a molecular graph (G) into a numeric value, i.e., *TI*(*G*) → *R*:

$$TI\left(G\right)=\sum_{u∼v}F\left(d_{u},d_{v}\right)$$

An important class of topological indices are degree-based topological indices, which are functions of the connectivity matrix of a molecular graph. The connectivity matrix for a molecule with n atoms is an *n x n*-matrix that designates a 1 when *u* ~ *v*, i.e., when atom u and v are bonded. These indices are summations over bonded atoms (*u* ~ *v*) where *d_u_* denotes the degree (number of bonds) of atom *u* and *d_v_* the degree of atom *v*.

The first topological index was the Randic index (Randic, 1975). It purports to characterize “molecular branching”. The Randic topological index takes on lowest values for linear molecules (like short chain fatty acids) and takes on the highest values (*n*/*2* for an *n* atom molecule) for molecules where all atoms are bonded to each other. The Randic topological index is a powerful predictor for drug related QSAR endpoints (Gutman, 2013).

$$R\left(G\right)=\sum_{u∼v}\frac{1}{\sqrt{d_{u}d_{v}}}$$

While some topological indices are strong predictors of chemical properties, their flexible nature has led to an incredible proliferation (Gutman, 2013). Unfortunately, the generation of large number of features with questionable causal relationships to QSAR endpoints leads to models that are overfit and impossible to interpret. Topological indices must be carefully considered before inclusion in any QSAR models.

#### 3.1.4 Dictionaries

Dictionary fingerprints indicate the presence or absence of different substructures or graph traversal results in a chemical. Dictionary fingerprints might specify a 6-carbon ring as a subgraph of interest, or they might ask whether more than 12 hydrogens are present in a chemical structure. Unlike hashed fingerprints, dictionary fingerprints define the subgraphs of interest before evaluating chemicals. This means they only require one pass over the given chemical library to generate a vector for each chemical.

Dictionaries seek to identify all the subgraphs of interest and as such they may miss important subgraphs particular to a given chemical set/endpoint. Dictionary fingerprints can be built for specific purposes; this sometimes involves a feature selection process wherein a dictionary is reduced to a smaller number of “important” features (Luechtefeld et al., 2015).

##### PubChem2D

The PubChem2D fingerprinter (Kim et al., 2016a) is a dictionary containing 7 “sections” with a combined total of 881 features. A PubChem2D fingerprint can be derived using the chemistry development kit (Willighagen et al., 2017). For example, a fingerprint for 1,4-pentanediamine contains 22 out of the 881 molecular graph PubChem2D features. Each section in PubChem2D represents a different kind of molecular graph feature^18^. These sections are described below.

##### Hierarchic element counts

These features simply look for the presence of different atoms. Individual atoms can have large impacts on molecular properties (i.e., the absence of any carbon atoms in a compound excludes it from normal organic chemistry). Examples include: ≥ 4 hydrogens, ≥ 8 hydrogens and ≥ 1 Hg. Note that the first two examples are not independent features. Feature independence can be a desirable property and is reviewed in Section 5.

##### ESSSR ring set

Extended smallest set of smallest rings (ESSSR) ring sets count the number of rings (defined as 3 or more atoms with a graph cycle) in a structure. Examples include ≥ 1 any ring size 3 or geq1 saturated or aromatic carbon-only ring size 3. Rings are important in cheminformatics due to their stability and relative inertness.

##### Simple atom pairs

Atom pairs identify the presence or absence of different pairs of atoms. Examples include: Li-H, C-F and N-H.

##### Simple atom neighbors

Simple atom neighbors identify the presence or absence of neighbors for a given atom (without regard to bond order). Simple atom neighbors use the ~ symbol to denote “any neighbor” and the ~ symbol to denote that the neighbor is part of a ring. Examples include C(~ C)(~ Si) and C(~ Cl)(:C)

##### Detailed atom neighborhoods

Like simple atom neighbors, except bond order is encoded as – (single), = (double), # (triple). Examples include C-C-C#C, S-S-C:C and N-C=N-C.

##### SMARTS patterns (simple and complex)

SMARTS (Smiles arbitrary target specification) are regular expressions for SMILES identifiers. The SMARTS language provides a syntax for matching patterns in SMILES strings^19^. For example, the SMARTS expression” [OH]c1ccccc1” captures all chemicals with a phenol ring.

### 3.2 Physics-based descriptors

Quantum and molecular mechanics based descriptors use computational chemistry to estimate molecular wave functions. The molecular wave function can be used to derive features describing electron density, charge, binding potential and more.

#### 3.2.1 Quantum mechanics

Quantum mechanical (QM) features are derived from the molecular wave function. While it is beyond the scope of this review to investigate the mathematics behind these features, it is important to understand their general descriptions and calculation. Molecular wave functions extend Schrödinger’s equation to describe many-bodied systems (molecules). A complete molecular wave function can be used to calculate electron densities and resulting electronic properties of molecules. Solving the molecular wave function is a computationally difficult task. QM solvers are divided between *ab initio* and semi-empirical approaches, where *ab initio* computation attempts to solve the molecular wave function from first principles and semi-empirical approaches are parameter-driven approaches with parameters fit by empirical data (Puzyn et al., 2008). Wave function solvers are an area of active research. Features derived from different solvers can be significantly different, but they tend to be biased in the same direction for different compounds (Thiel, 2014). Like QSARS, semi-empirical solvers sometimes incorporate machine learning in parameter estimation (Thiel, 2014). QSARs using features based on these solvers must be careful of compounding errors wherein bias/error in modeled features are magnified by their use in statistical models.

A brief description of the categories of QM descriptors grouped by Karelson et al. (1996) is given below:

– *Charges* – Charge descriptors measure aggregate statistics for atomic charges in a molecule. The sum of squares of all atomic charges is one example (Buydens et al., 1983). All chemical interactions are either through electrostatic (polar) interactions or covalent (orbital) interactions. Atomic charge is a major governing feature for such interactions.
– *HOMO and LUMO energies* – When atoms bond to form molecules, their atomic orbitals combine to form molecular orbitals. Molecular orbitals describe the location and density of electrons in a molecule. Frontier orbital theory describes reaction mechanisms primarily as interactions between the HOMO (highest unoccupied molecular orbital) and LUMO (lowest unoccupied molecular orbital) of molecules (Fukui et al., 1952). HOMOs and LUMOs are not numeric features, they are distributions of electron density. HOMOs and LUMOs can be used to derive related features like the energy of each and the difference in energies between each.
– *Atomic and molecular polarizabilities* – Polarizability measures the potential for an atom or molecule to form a dipole (separation of positive and negative charges). Polarizability is an important property of all molecules as it is a strong predictor of how the molecule will behave with other non-polar or polar molecules. Polar surface area (PSA) is a sum of the contribution of polar atoms to the total surface area of a molecule and is a powerful predictor of endpoints such as intestinal absorption, blood-brain barrier and more (Prasanna and Doerksen, 2009).

QM approaches to QSARs are extremely powerful but also susceptible to misuse. Several packages, such as dragon descriptors^20^, generate thousands of features derived from QM calculations. Models built from these features are vulnerable to overfitting due to the large number of features and difficulty in interpreting features.

### 3.3 Comparative molecular field analysis (CoMFA) and simulated QSARs

While not covered in depth here, some approaches to computational models perform direct simulations on chemical structures. Rather than building statistical models from chemical descriptors, these models create a virtual 3-dimensional physical model of the chemicals and receptors. Models of receptor-ligand binding can simulate physical interactions between molecules and protein receptors. One important example of this approach is comparative molecular field analysis (CoMFA) (Cramer et al., 1988). In a CoMFA analysis, a set of compounds known to interact with the same receptor are selected. A training subset is selected and potential pharmacophores (functional groups that interact with the receptor) are generated via superposition of the training set. CoMFA approaches can become much more complex, but in general they operate by virtual manipulation of 3-dimensional structures (with charges).

## 4 Toxicological targets

QSARs can be used to address diverse targets in toxicology: These targets include (but are not limited to) physicochemical properties, environmental effects, and human health effects. To build a QSAR, some form of reference data is needed, usually in the form of an experimental observation. When the desired endpoint is difficult, expensive or dangerous to generate directly, an experimental surrogate or biomarkers for the endpoint can be used. For example, the LLNA (Luechtefeld et al., 2016a) is performed on mice and used as a stand-in for the human patch test to evaluate human skin sensitization (Gerberick et al., 2001).

### 4.1 Problems to address a well-defined endpoint

The OECD QSAR guidelines suggest that QSARs should have a well-defined endpoint. Well-defined endpoints clearly identify the experimental target of a QSAR and minimize ambiguity in the assignment of target values to chemicals. There are a few problems, however:

#### 4.1.1 Identifier based ambiguity

Publications often define QSAR targets using natural language, for example: “acute aquatic toxicity” (Lozano et al., 2010), “postmortem redistribution” (Giaginis et al., 2014) or ”placental clearance and transfer” (Hewitt et al., 2007). These names vary from highly descriptive to vague. The uncontrolled nature of natural language names results in multiple names for the same target and ambiguous names that could refer to multiple targets. This problem is partially addressed by agencies that define unique identifiers for QSAR targets.

#### 4.1.2 PubChem bioassays capturing activity across multiple targets

PubChem creates a unique identifier for bioassays (Wang et al., 2010), which refers to a specific screening test and the values generated thereof. Even experimental endpoints identified via alphanumeric identifiers can be difficult to define as screening assays capture activity across multiple targets. This multi-target identification leads to problems. If a QSAR models this assay, does it also model assays that measure activity against a subset of the targets of this assay?

#### 4.1.3 OECD guidelines result in variability

The OECD guideline identifier provides detailed descriptions of different experimental procedures. OECD TG 407, for example, is the OECD guideline for the test “Repeated Dose 28-Day Oral Toxicity Study in Rodents” (OECD, 2008). This is a 13-page guideline, which has a revision history (one major change in 1995) that covers important initial considerations, the principle of the test, and a detailed description of the method. These guidelines describe in some detail how to interpret the results of guideline studies and what parameters may be changed for the study. Parameter choices for TG 407 include selection of animal species (preferably rats, but any rodent with sufficient justification.) The ability to change parameter selection for guidelines allows greater experimental variance. OECD guideline outcomes make excellent QSAR targets. They are the result of a well-defined study with careful instructions on how to measure the results. However, even using results from OECD guidelines studies is not without peril. In a study of the consistency of the Draize rabbit eye irritation test (another OECD guideline study), we found a low rate of concordance between studies (Luechtefeld et al., 2016b). Variance in repeated guideline study results can be large and bias can exist between labs performing experiments.

#### 4.1.4 UN GHS hazards and variety of assays used to determine them

The United Nations Global Harmonized System (GHS) of Classification and Labelling of Chemicals seeks to improve communication of chemical hazards. UN GHS hazards are identified by an alpha numeric such as H317 – May cause allergic skin reaction. GHS hazards are a communication tool rather than a means to identify a specific procedure for determining a chemical hazard. Multiple different kinds of studies can be aimed at determining the same UN GHS hazard. The mouse LLNA, guinea pig maximization test (GPMT) and Buehler test are all examples of animal models for H317 (Luechtefeld et al., 2016a). It is up to regulatory agencies to specify preferred tests.

Classification and labelling datasets, like the one generated by the European Chemicals Agency from company submissions in service to REACH, catalogue UN GHS hazards for large numbers of chemicals. These databases can be powerful resources for computational model target data owing to their size and consistency. They are particularly data-rich for the topical and acute hazards (Luechtefeld et al., 2016a–d). However, the variability of methods used to classify different chemicals leads to the potential for bias in models built from different experimental methods. Commercial and regulatory entities sometimes use UN GHS hazards to define policies. Safety data sheets are one example of this commercial and regulatory use (Winder et al., 2005). When defining policies based on these labels, the question of when to perform which tests has not been well answered. Some UN GHS hazards are redundant, correlated to others, or conflict with others (Wilrich et al., 2017). These questions are being addressed by integrated testing strategies.

### 4.2 Integrated testing

QSAR performance can be improved via the integration of many endpoints. The concept of a single, well-defined endpoint is a limiting concept. For instance, some of the UN GHS hazards are redundant and some are mutually exclusive. A chemical that is pyrophoric (UN GHS Hazard H250) is also a flammable liquid (UN GHS Hazard H228). While endpoint relationships create challenges for testing – i.e., should testing be required when the endpoint can be derived from a redundant existing label? (Wilrich et al., 2017) – they create opportunities for QSARs. We have discussed the opportunities of integrated testing earlier (Hartung et al., 2013; Rovida et al., 2015).

One form of integrated testing is weight of evidence (WoE) (Linkov et al., 2015). In this paradigm, multiple different experiments that purport to model a given endpoint are combined to give a stronger classification. When WoE is used in a quantitative manner, it typically refers to a majority vote or other simple aggregation of multiple test results (Weed, 2005).

Adverse outcome pathways (AOP) encode logical relationships between biological events that end in one “adverse outcome” like skin sensitization, cholestasis, liver fibrosis, etc. (MacKay et al., 2013). An AOP provides a conceptual framework on which integrated testing strategies can be built (Tollefsen et al., 2014). It consists of (1) molecular initiating event, (2) intermediate key events and (3) an adverse outcome. The OECD definition of an AOP for skin sensitization (OECD, 2014) states that a skin sensitizer must (1) penetrate the skin and be metabolized, (2) be an electrophilic substance, (3) covalently interact with proteins, (4) interact with dendritic cells and keratinocytes and (5) cause the proliferation of activated T-cells in the lymph node. The ability of a chemical to pass each stage of this AOP can be tested in independent tests.

### 4.3 Probabilistic graphical models

Probabilistic graphical models (PGM) enable statistical models to operate on relationships between known values. These are discussed again in Section 5, but are important in the context of integrated testing strategies. PGMs can use adverse outcome pathways to combine the result of different endpoints in a stronger way than typical WoE (Jaworska, 2016).

One important example of PGM-based integrated testing strategies is the Bayesian Network Integrated Testing Strategy (ITS-3), which integrates a QSAR with multiple *in vitro* assays to model skin sensitization (Jaworska et al., 2015). The ITS-3 probabilistic graphical model is used for the mouse LLNA, an experimental model for skin sensitization. Each of the variables in this model is an *in vitro*, *in silico* or chemical property. Arrows between nodes denote probabilistic relationships. The model contains “hidden” nodes that are not directly observed but rather cluster observations from attached variables. *In vitro* variables include KEC3 and KEC1.5, which are results of KeratinoSens reactivity assays. The model uses DPRALys and DPRACys, which are direct peptide reactivity assays for cysteine and lysine. These variables map directly to the adverse outcome pathway for skin sensitization. The combination of AOP and PGM is a powerful method for improving upon WoE integrated testing strategies. In addition to modeling endpoint results, they can be used to make estimates using incomplete information and thus enable sequential testing (below).

While not yet commonly used, sequential testing strategies promise to reduce the experimental cost of modeling QSAR endpoints. Models like the above-mentioned probabilistic graphical models can be used to determine optimal testing strategies that seek to achieve some required level of accuracy for an endpoint while minimizing the cost and number of tests. For instance, if a chemical is negative in several reactivity assays, it may be unnecessary to perform immunological assays such as the h-CLAT (an immunological *in vitro* assay in the Jaworska model).

## 5 Algorithms to predict toxicological properties of substances

This chapter reviews some of the main concepts behind algorithms used in QSAR development. It is not a deep dive into any statistical or machine learning approaches; rather it gives a high-level view of the important machine learning ideas relevant to computational toxicology models. James Governor (Principal Analyst and founder of RedMonk) nicely said “*Data matures like wine, applications like fish*” – in this sense we will not look for a snapshot of the currently available software but discuss some principles and problems.

### 5.1 Classification and regression

The primary goal of most QSARs is to use features (mostly structural) to model endpoints. These endpoints are typically binary, numeric or categorical.

Binary toxicological QSARs classify chemicals as true or false for a given property. Many of these QSARs attempt to classify chemicals as hazardous *vs* not hazardous. For example, the article *Automatic knowledge extraction from chemical structures: the case of mutagenicity prediction* (Ferrari et al., 2013) evaluates a mutagenicity classifier (mutagen *vs* non-mutagen) called SARpy that identifies predictive structural fragments directly from SMILES strings.

Numeric toxicological QSARs often identify a parameter related to dose-response. An example is the Japanese Ministry of Environment’s Kashinhou tool for ecotoxicity (KATE) system, which evaluates the concentration which kills 50 percent of daphnia (LC_50_) (Furuhama et al., 2011). KATE is a linear equation relating chemical octanol/water partition coefficient (log P) with aquatic toxicity. Some analyses include a chemical categorization step for identifying domain of applicability (Furuhama et al., 2011).

Categorical QSARs classify chemicals as having one of several values for a given property. Categorical QSARs can be nominal (name only) or ordinal (ordered names).

Nominal categorical QSARs classify chemicals by some named property. The semantic link association prediction (SLAP) algorithm uses semantic links, i.e., chemical A → is similar to → chemical B or chemical B → treats → disease A to model drug-drug interactions, drug-disease relationships and other properties of chemicals (Chen et al., 2012). Nominal QSARs can often be built from multiple binary QSARs, which each model the truth of one value for the named property (i.e., chemical A treats hypertension).

Ordinal QSARs impose an order on the named values for a property. Chemical potency, such as weak, medium, and strong skin sensitizers, is one one such ordinal category. The Jaworska Bayesian network predicts skin sensitization potency by using features like *in vitro* assays, *in silico* results (results from other models), and physical properties (Jaworska, 2016).

The above examples are all statistical models. It is also possible to create classification models from expert knowledge. These are termed expert systems. *Expert systems* can be combined with statistical techniques to fit parameters in an otherwise human-defined rule set. Verma and Matthews (2015) publication *An* in silico *expert system for the identification of eye irritants* does just this by deriving rules for eye irritation using chemical hydrophobicity, molecular weight and several other physical properties. One example of a classification algorithm is given below. Many textbooks describing the creation of multitudes of classification algorithms.

### 5.2 Classification trees

Classification trees provide an excellent example of binary/categorical classifiers. Consider a dataset classifying chemicals as positive or negative for some hazard. Classification trees iteratively divide the data set as follows:

1. Create a measure of the “fitness” of a collection of data. A common choice is entropy or Gini coefficient. This fitness function will prefer datasets where all elements have the same label (all positive or negative).
2. Collect all data and measure fitness.
3. Choose a feature and separate the dataset into child datasets where all elements in a child have the same value for the chosen features. For example, if the chosen feature were “electrophile”, then dividing the dataset according to this feature would result in two datasets: one with all electrophiles and one with no electrophiles.
4. Measure the average fitness of the child datasets for the chosen features. Find the difference between this value and the fitness of the parent dataset.
5. Repeat steps (3) and (4) for each feature in the dataset and choose the “fittest” features
6. Repeat steps (2) through (5) until some terminal state.

Classification trees are one of the simplest approaches to supervised learning. Step (5) above is an example of a greedy search. It is sometimes possible to do better using non-greedy searches, but depending on the model used, the search space can be very large. For a dataset where all features are binary, 2^n^ different trees can be created.

Toxtree is one example of a classification tree used in a public computational model. It uses “reactivity alerts” as features that identify chemicals that have the potential to be reactants in chemical reactions believed to be relevant to skin sensitization.

**Fig. 9:**
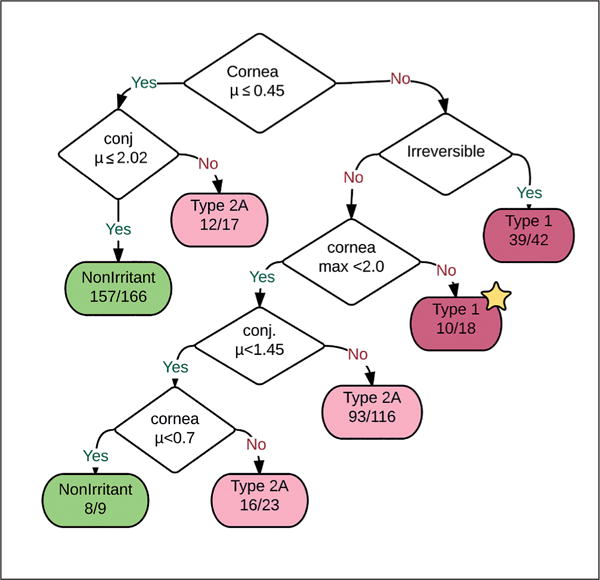
A decision tree built from a large number of Draize eye irritation tests In the Draize test, several features of the exposed animal eye are measured including cornea damage, conjunctivae damage, irreversibility of the observed damage and a few others. The decision tree built from the hundreds of Draize eye irritation tests closely matches the OECD guidelines (reproduced from Luechtefeld et al., 2016b).

In our recent publication analyzing Draize eye irritation testing and its prediction by mining REACH data (Luechtefeld et al., 2016b), a decision tree (reproduced in Fig. 9) is used to demonstrate that the guidelines for Draize eye irritation tests (Hartung et al., 2010) are in fact followed in the literature.

### 5.3 The problem of overfitting

The careful reader will note that, for the aforementioned decision tree example, if enough features are given for a dataset, spurious features may be chosen in the creation of a decision tree. Even random features will occasionally divide datasets into desirable child datasets. This realization is the root of overfitting. Or in other words: “*Torture the data, and it will confess to anything*.” – Ronald Coase (British economist and author, 1910-2013).

Overfitting is a pernicious problem in chemical modeling. Most toxicological datasets are extremely small relative to the chemical universe. Additionally, it is possible to generate thousands of features based on structure alone. These two features combined make it possible to build models that perform very well on the training set but then fail to perform well when given novel data.

Molecular interactions drive most toxicological phenomena. The interactions can be complex and sensitive to small changes in chemical structure. Because of this, models that capture relationships between certain chemical features and observed endpoints may only be valid for the training sets on which they are built.

There are methods to combat overfitting. Most involve hiding some portion of the existing data from the machine learning algorithm and then testing the algorithm on the hidden data. While this can be helpful, it will still fail when very large numbers of features are used.

The problem of overfitting can be combatted by using features that are well understood by the modeler, by using cross-validation or other data hiding methods in model evaluation, and ultimately by using the largest data sets possible. The OECD guidelines for QSAR development state that algorithms should have a mechanistic interpretation if possible. Mechanistic interpretations further guard against overfitting. Some machine learning models can be difficult to interrogate, but careful dataset building (including only relevant features) and the use of data visualization tools can help to provide a rational mechanistic hypothesis for model success.

### 5.4 Domain of applicability

The OECD guidelines for QSARs state that models should have a defined domain of applicability (DoA). The DoA for a model’s classifications is the set of chemicals for which those classifications are likely to work well. DoA is important for the creation, validation and application of QSARs (Roy, 2017). In development, DoA informs modelers on the kinds of data that should be used and the set of chemicals. Picking a narrow DoA may strengthen the predictive value of a model, but reduce its use cases. During validation, modelers should define their DoA to those chemicals they expect the model to perform well on. This has the effect of more accurately evaluating model performance. Finally, modelers must be careful that their models are not misused on chemicals that are not part of their DoA.

Some models are designed to tackle a specific DoA and explicitly define their DoA. A few examples of QSAR publications with varying strictness of definition for DoA include:

1. *Development of a novel mathematical model using a group contribution method for prediction of ionic liquid toxicities:* Ismail Hossain et al. (2011) built a mathematical model focused on ionic liquids on the whole. This is a broadly defined DoA.
2. *Therapeutic index modeling and predictive QSAR of novel thiazolidin4-one analogs against Toxoplasma gondii:* Asdollahi-Baboli and Mani-Varnosfaderani (2015) built a QSAR specifically targeted at thiazolidin-4-one analogs. This is a very targeted and narrow DoA.

Some models implicitly define their DoA by the chemicals used in training. There are a few methods for defining applicability domain on learned models. Typically, these methods define a chemical similarity metric (or distance metric) and define chemicals as part of a DoA when they are sufficiently similar to chemicals used in training (Nikolova and Jaworska, 2003). Chemical similarity is a complex topic of its own. Both chemical similarity and DoA are areas of enormous development needs and opportunities.

## 6 Conclusions

The advent of big data in toxicology drives new approaches for the prediction of hazard (Hartung, 2016). To a large extent, it changes the common paradigm of a hypothesis-driven research, as it collects data first and then tries to mine them. We might call this a Sherlock Holmes approach, as Arthur Conan Doyle stated “*It is a capital mistake to theorize before one has data. Insensibly, one begins to twist the facts to suit theories, instead of theories to suit facts*”. Here, the basics of these approaches have been discussed.

This is not arguing for a blind belief in such statistical evaluation of data. To quote Alvin Toffler (1928-2016): “*You can use all the quantitative data you can get, but you still have to distrust it and use your own intelligence and judgment*”. We need to strike the right balance between the evidence-based and the eminence-based approaches in our field. We have stressed with this article once again the opportunities of an evidence-based toxicology (Hartung, 2009; Hoffmann and Hartung, 2006), here by proper mining of chemical and toxicological knowledge of the past. In fact, evidence-based toxicology is about treating data properly and about treating as much of it as we can possibly get. Because, as Swedish mathematician and writer Andrejs Dunkels (1939-1998) phrased it: “*It’s easy to lie with statistics. It’s hard to tell the truth without statistics*”.

## Figures and Tables

**Fig. 7:**
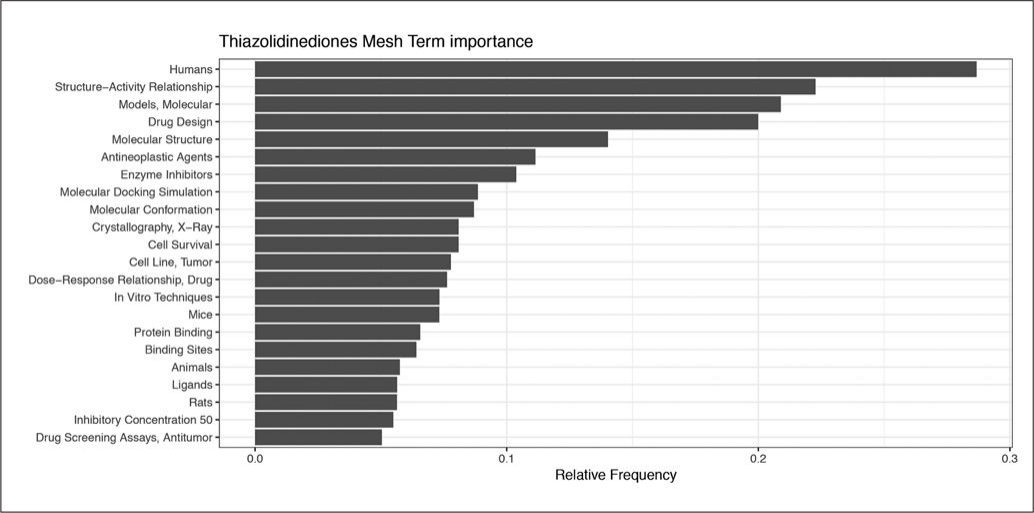
MeSH term importance for thiazolidinediones QSARs relative to QSARs in general

**Table 1:** Number of Github repositories for queries pertaining to QSAR development Github.com was queried with the terms in the first column and the number of repositories counted.

Query	Github repositories
machine learning	63,989
statistics	20,146
chemistry	2,189
biology	1,924
cheminformatics	128
QSAR	95

## Footnotes

1 https://pubchemblog.ncbi.nlm.nih.gov/2014/06/19/

2 https://www.ebi.ac.uk/chembl/

3 https://www.bindingdb.org/

4 http://cheminfov.informatics.indiana.edu:8080/c2b2r/

5 https://github.com

6 https://en.wikipedia.org/w/index.php?title=PubChem&oldid=801836176

7 http://www.oecd.org/env/ehs/risk-assessment/37849783.pdf (last accessed 11 Oct 2017)

8 https://meshb.nlm.nih.gov/record/ui?ui=D021281

9 www.daylight.com/dayhtml/doc/theory/theory.smiles.html

10 https://www.cas.org/content/chemical-substances/faqs

11 https://en.wikipedia.org/w/index.php?title=Chemical_table_file&oldid=791936248

12 http://www.inchi-trust.org/technical-faq/

13 https://www.drugbank.ca

14 https://pubchem.ncbi.nlm.nih.gov/search/search.cgi

15 https://www.ebi.ac.uk/chebi/

16 https://www.ncbi.nlm.nih.gov/mesh/68045162

17 https://meshb.nlm.nih.gov/record/ui?ui=D021281

18 ftp://ftp.ncbi.nlm.nih.gov/pubchem/specifications/pubchem_fingerprints.txt

19 http://www.daylight.com/dayhtml/doc/theory/theory.smarts.html

20 http://www.talete.mi.it/products/dragon_molecular_descriptors.htm
